# Olivine and dissolved alkalinity trigger different bacterial community shifts in water and oyster gills: insights from a mesocosm experiment

**DOI:** 10.3389/frmbi.2025.1659695

**Published:** 2025-09-26

**Authors:** Dominik Antoni, Marco Rump, Gunnar Gerdts

**Affiliations:** Alfred-Wegener-Institut Helmholtz-Zentrum für Polar- und Meeresforschung, Biologische Anstalt Helgoland, Helgoland, Germany

**Keywords:** ocean alkalinity enhancement, negative emission technology, environmental microbiology, olivine, ecotoxicology, mesocosm experiment, metabarcoding

## Abstract

Ocean Alkalinity Enhancement (OAE) is a proposed marine carbon dioxide removal strategy that increases seawater buffering capacity and CO_2_ uptake through the addition of alkaline substances. While OAE shows promise as a climate mitigation tool, its ecological implications remain poorly understood, particularly regarding microbial communities. This paper provides a risk assessment of two different OAE strategies: alkalization with olivine and alkalization with addition of dissolved sodium hydroxide (NaOH). With a mesocosm experiment designed to simulate coastal OAE application, European flat oysters (*Ostrea edulis)* were chronically exposed to alkalinity-enhanced seawater at two concentrations (250 and 500 µmol·L^-^¹) derived either from olivine weathering or addition with NaOH. The bacterial community composition of both alkalization types was assessed with amplicon sequencing of the 16S rRNA gene and ecotoxicological impacts were compared to a non-alkalized control. The sampling strategy included samples of the treated waters and the gill microbiome of *Ostrea edulis.* Our results show that the alkalization type was the primary driver of microbial shifts in the bacterial community of the water samples. Olivine treatments caused distinct taxonomic changes, including an increase in Gammaproteobacteria and Flavobacteriales and a marked decline in Alphaproteobacteria and SAR11 clade. Olivine-treated waters showed reduced richness and evenness. In contrast, dissolved alkalinity treatments produced minimal changes compared to untreated controls. The analysis of the oyster gill microbiome detected a response that was stronger influenced by alkalinity concentration than by alkalization type. Notably, high-alkalinity olivine treatments favored potentially pathogenic *Vibrios*. Together, these findings highlight that OAE method selection significantly influences bacterial community composition in both marine and host-associated microbiomes. In our experiment, olivine-based OAE posed a greater environmental risk than dissolved OAE. Our study provides insights on the impact of different OAE scenarios, representing a first step toward future field trials and applications.

## 1 Introduction

Anthropogenic fossil fuel combustion increased atmospheric CO_2_ concentrations from 280 ppm in preindustrial times to 424 ppm in 2024 (Friedlingstein et al., 2024), a level not seen for approximately 3 million years (Rae et al., 2021). This 50% rise has contributed to a global temperature increase of approximately 1 °C in the atmosphere and 0.88 °C in the upper ocean (Allan et al., 2023; Stocker, 2014).

In addition to causing global warming, rising atmospheric CO_2_ contributes to ocean acidification. The ocean is a major carbon sink, as it absorbs atmospheric CO_2_ which forms carbonic acid upon dissolution. This reaction increases hydrogen ion concentration, thereby lowering the pH of the. While this uptake helps buffer atmospheric CO_2_ levels, it leads to ocean acidification (Turley and Findlay, 2016). corresponding to a pH decline of at least 0.1 units (Metzl et al., 2025; Raven et al., 2005). Current models project that ocean acidification and global warming are projected to continue, with some scenarios projecting global temperature increases exceeding 4 °C above preindustrial levels, along with an additional pH decline of up to 0.3 units (Jiang et al., 2023).

Global warming and ocean acidification have severe ecological consequences and also drive extreme weather events, which in turn cause widespread societal and economic damage (Weiskopf et al., 2020). To address the impacts of climate change, global leaders established the Paris Agreement in 2015. The aim of the agreement is to limit global CO_2_ emissions and restrict warming to the defined climate goals of 1.5 °C or at most 2 °C above preindustrial times to minimize damage to ecosystems and human populations (Maibach et al., 2019; Rhodes, 2016).

Along with limiting CO_2_ emissions, there is a need to remove atmospheric CO_2_ through Negative Emission Technologies (NETs). NETs are defined as anthropogenic processes that remove more CO_2_ from the atmosphere than they emit (Minx et al., 2018). Over 80% of the scenarios that limited warming to 2 °C included NETs in the second half of the century. With accelerating warming and a growing NETs market, research increasingly focuses on their CO_2_ removal potential, as well as on feasibility, environmental risks, and monitoring, reporting, and verification (MRV) requirements (Yao and Zhang, 2025).

One such NET is Ocean Alkalinity Enhancement (OAE). Alkalinity refers to a water body’s capacity to buffer pH changes caused by acid input. Chemically, it is defined as the excess of proton acceptors over proton donors in solution (Gran, 1952; Montserrat et al., 2017). OAE is a NET mitigates ocean acidification by buffering carbonic acid while simultaneously enhancing the ocean’s ability to absorb CO_2_ (Fuss et al., 2018). OAE offers a broad range of potential applications (1) Electrochemical methods can split seawater into acidic and basic compartments. This can be used to remove the acidity and thus increase the total alkalinity of a water body (Ringham et al., 2024); (2) Enhanced rock weathering, which involves applying finely ground minerals (e.g., olivine) to promote rapid weathering (e.g., olivine) in high-energy coastal environments to accelerate dissolution and CO_2_ uptake (Schuiling, 2017); and (3) Direct alkaline addition, where substances such as sodium hydroxide (NaOH) or calcium hydroxide (Ca(OH)_2_) are added to seawater to increase alkalinity immediately (Hartmann et al., 2022; Marín-Samper et al., 2024). Despite their potential for large-scale CO_2_ sequestration, the environmental risks of these methods remain poorly understood (Meysman and Montserrat, 2017; Riebesell et al., 2023; Vicca et al., 2022).

Different OAE methods can have distinct ecological consequences. OAE may lead to localized pH fluctuations that affect sensitive marine organisms such as coral larvae and other planktonic organisms dependent on stable pH conditions (Nakamura et al., 2011). Shifts in pH caused by OAE could disrupt carbonate chemistry, affecting calcifying organisms such as corals, shellfish, and coccolithophores (Lehmann and Bach, 2025; Thomsen et al., 2015). Furthermore, changes in alkalinity could alter primary production by inducing CO_2_ limitation in seawater (Harms et al., 2024; Suessle et al., 2025). Such changes may cascade through the food web, leading to complex and unforeseen ecological effects. Enhanced rock weathering with minerals such as olivine can release trace metals from natural impurities into the marine environment. Heavy metals like nickel and chromium, for example, can pose toxicity risks to marine life, while iron can fertilize habitats that are iron-limited (Flipkens et al., 2021). These uncertainties highlight the need for comprehensive environmental risk assessments before large-scale deployment of any OAE.

In recent years, research on bacterial communities has gained attention. With the increasing affordability of Next-Generation Sequencing (NGS), environmental barcoding is frequently used for characterizing bacterial diversity and its ecological relevance. The Earth Microbiome Project is an example of a global initiative that provides standardized protocols for assessing bacterial communities (Gilbert et al., 2018). In ecotoxicological studies, barcoding is used to assess the effects on microbial communities of pollutants like heavy metals (Kou et al., 2018), microplastics (Song et al., 2022) and chemical contaminants (Salerno et al., 2018).

Certain bacterial taxonomic groups are linked to specific processes in the water which can either contribute to carbon sequestration or indicate environmental pollution. Proteobacteria, one of the largest bacterial phyla, are subdivided into Alpha- and Gammaproteobacteria, among others. Alphaproteobacteria are more abundant in oligotrophic waters, while Gammaproteobacteria thrive in nutrient-rich and polluted environments, often harboring opportunistic pathogens such as species from the *Vibrio* genus (Fontaine et al., 2023; Newton et al., 2011). Specific bacterial taxa, including Gammaproteobacteria and Actinobacteria, often show increased relative abundance in contaminated environments, serving as indicators of pollution (Moreira et al., 2023). Furthermore, marine bacteria play essential roles in nutrient cycling and contribute to biogeochemical processes that sustain marine ecosystems. For example, members of the Roseobacter clade participate in multiple carbon fixation pathways (Brinkhoff et al., 2008; Tang et al., 2016). Certain nitrogen-fixing cyanobacteria form obligate symbioses with photosynthetic eukaryotes, supplying fixed nitrogen in exchange for organic carbon, thereby supporting primary production and enhancing carbon sequestration (Zehr and Capone, 2024). Bacteria from the genus *Marinobacter*, facilitate marine snow formation, a process in which algae aggregate and sink as organic detritus into the deep ocean, where carbon is stored for long timescales (Gunasekera et al., 2022).

OAE applications are more likely in coastal zones, where infrastructure and high-energy wave activity can be harnessed to facilitate mineral dissolution and alkalinity dilution (Schuiling, 2017). In coastal environments, oysters play a key ecological role as filter feeders and hold significant economic value. They improve water quality and provide habitat for diverse marine species (Van In and O'Connor, 2024). Oysters are valuable model organisms for assessing ecotoxicological effects of OAE (Barros et al., 2013). It is hypothesized that OAE enhances calcification in marine organisms by facilitating carbonate formation (Bednaršek et al., 2025). However, the ecological consequences of OAE on oyster require experimental validation.

One of the main challenges in OAE research is the restrictive regulatory framework. Field trials, which are essential for assessing environmental impacts, are rarely conducted due to unclear legal frameworks (Steenkamp and Webb, 2023). As a result, OAE research is currently limited to mesocosm and laboratory-scale studies using model organisms.

We aim to evaluate two different OAE materials by investigating taxonomic shifts in bacterial communities, using a mesocosm experiment. We sampled bacterial communities from the alkalized water and from the gills of European flat oysters (*Ostrea edulis*) that were exposed to these waters. We compared a mineral dissolution strategy (weathered olivine sand) with a dissolved alkalinity approach using sodium hydroxide (NaOH) augmented with calcium chloride (CaCl_2_), the latter simulating dissolved limestone.

We hypothesize that alkalinity addition will have a concentration-dependent effect on the beta diversity of the bacterial communities. Because our alkalinity treatments exceed those of previous olivine studies that reported changes in the water microbiome (Ren et al., 2022), we also expect to observe statistically significant microbial shifts. Previous studies using comparable dissolved alkalinity treatments observed only marginal effects on marine bacterial communities (Antoni et al., 2025). Therefore, we anticipate only minor shifts in microbial community composition in the dissolved alkalinity treatments relative to the olivine treatments.

By assessing the ecotoxicological impacts on both bacteria and oysters, this study addresses a key knowledge gap regarding the risks associated with different OAE materials. As a preliminary step, our findings may support future field trials and OAE deployment by addressing key questions about the safety of different materials and implementation strategies (Dupont and Metian, 2023).

## 2 Methods

### 2.1 Mesocosm facility

The mesocosm setup used in this experiment is described in detail by Mackay-Roberts et al. (2024). Brief description of important parameters: The mesocosm facility is located at the Alfred Wegener Institute research station on Helgoland, Germany (Dummermuth et al., 2023). It is a benthic mesocosm system, with each unit consisting of a 570-liter polypropylene exposure tank and a 70-liter polyethylene mixing tank. Seawater supply is established with water from the North Sea, pumped from 2–4 m depth, passing through a 340-m³ settlement tank (42-hour retention time) for sediment reduction and a 56-m³ header tank (6-hour retention time). Each mesocosm includes internal recirculation between the exposure and mixing tanks. The entire water volume of the mesocosms is recirculated every eight minutes and mixed via a spray bar system.

### 2.2 Water and oyster descriptions

The seawater is well studied and monitored through the Helgoland Roads long-term observational data series, which includes physicochemical measurements such as salinity, alkalinity, and temperature (Wiltshire et al., 2010). The general marine microbiome and the site’s seasonal succession are also well documented (Teeling et al., 2016).

European flat oysters (*Ostrea edulis*) used in this study were originally obtained as seed from a commercial hatchery (Seasalter, Walney, UK). The batch originated from a population cultivated by the PROCEED project for *Ostrea edulis* restoration in the North Sea (Pogoda et al., 2024). All oysters were two years old and belonged to the same size class (>4 cm in length). Prior to the experiment, the oysters were deployed in the North Sea for one year in baskets mounted on landers. Mesocosms were inspected daily, and dead oysters were removed and recorded to monitor the mortality rate.

### 2.3 Experiment setup and treatment description

**Figure 1:**
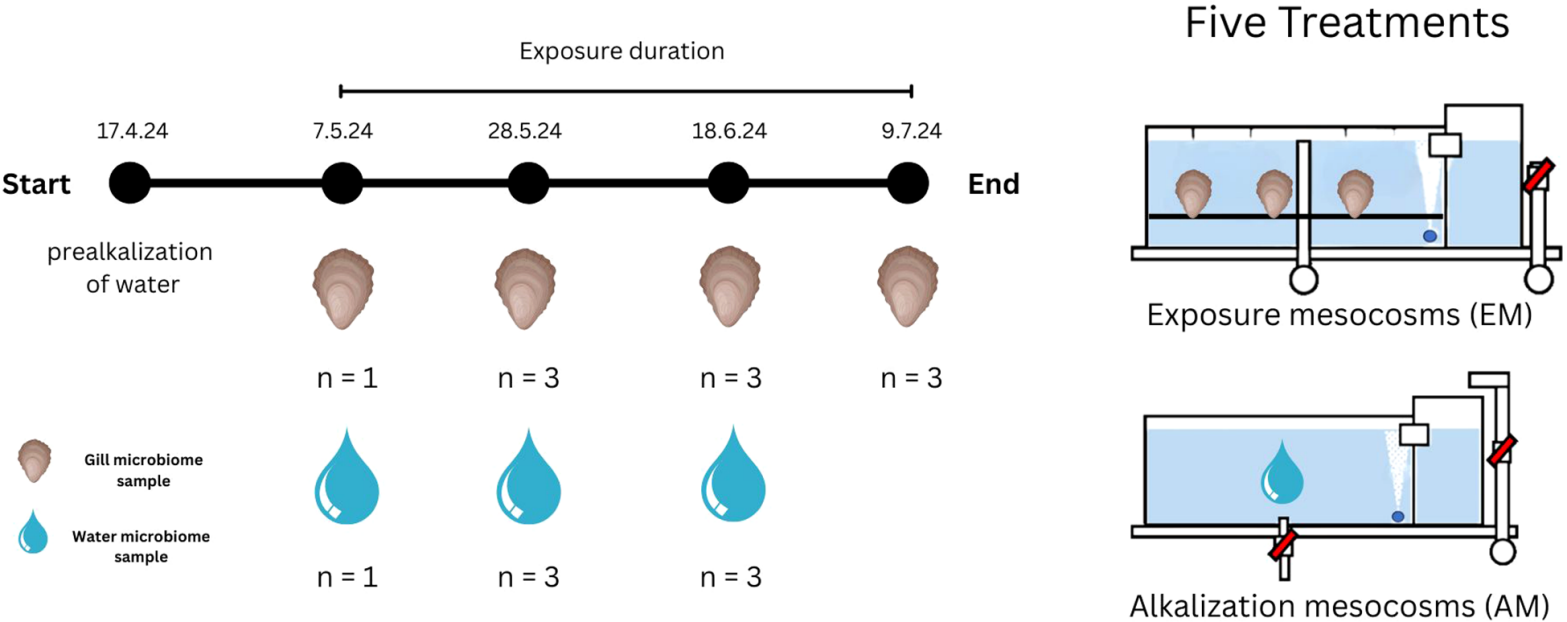
Sampling scheme and setup. Sampling was conducted every third week of the experiment. The experimental conditions included 5 treatments, each with two mesocosms. Water microbiome samples were taken from the alkalization mesocosms. These Mesocosms did not contain any oysters and were used to alkalize the water to the desired alkalinity levels. Gill microbiome samples were taken from the exposure mesocosms. The dates marked under the text “exposure duration” show the dates of the sampling events. Exposure lasted for 63 days. The number of replicate samples taken from each mesocosm is specified by “n”.

The mesocosms were filled with seawater filtered through a 100 µm mesh-sized nylon filter (SF-Filter GmbH, Bachenbülach). The experiment began on the 17^th^ of April 2024 with the onset of alkalization and ended on the 9^th^ of July 2024 (**Figure 1**). Ten mesocosms were used in total. Each treatment used two mesocosms: one alkalization mesocosm (AM) and one exposure mesocosm (EM). After each alkalization period, water from the AM was pumped into the corresponding EM. The EMs were used to expose *Ostrea edulis* to the alkalized water. The EMs of each treatment had a total of 60 oysters in them. While oysters were being exposed in the EMs, the AMs prepared water for the next cycle, enabling a water exchange but chronic alkalinity exposure. This design resulted in four exposure periods, each lasting three weeks.

The experiment included five treatments. The treatments consisted of one control with no alkalinity addition and two types of alkalinity enhancement (weathered olivine and dissolved NaOH), each applied at two target concentrations (250 and 500 µmol·L^-^¹). The olivine used was AFS 50. It is a product of the company Sibelco and is mined in Åheim, Norway. It consists of 93 mol% forsterite and 7 mol% fayalite, with a dominant grain size range of 0.125–0.250 mm. Alkalinity enhancement with olivine was achieved by mechanically weathering it in fluidized bed filters (FBF) (FB 5000, Aquamedic) as weathering reactors (See picture in **Supplementary Figure 1**). To reach the two target concentrations, one FBF was filled with 5 kg of olivine (250 µmol·L^-^¹) and the other with 10 kg (500 µmol·L^-^¹). The alkalization lasted for three weeks. The FBFs were used to minimize the intrusion of olivine particles into the mesocosms. For the dissolved alkalinity treatments, each mesocosm received either 5.40 g NaOH and 9.93 g CaCl_2_·2H_2_O (for 250 µmol·L^-^¹) or 10.81 g NaOH and 19.86 g CaCl_2_·2H_2_O (for 500 µmol·L^-^¹).

Water in the mesocosms was continuously bubbled with air to ensure oxygen saturation for the oysters. The bubbling was necessary for oyster survival, but it resulted in equilibrate OAE as an artefact of the methodology, since it equilibrated the water with surrounding CO2. Oysters were fed 25 mL of Shellfish Diet 1800 (Reed Mariculture Inc., Campbell, CA, USA) every other day, following the manufacturer’s dry weight feeding guidelines.

### 2.4 Water chemistry measurements

**Figure 2:**
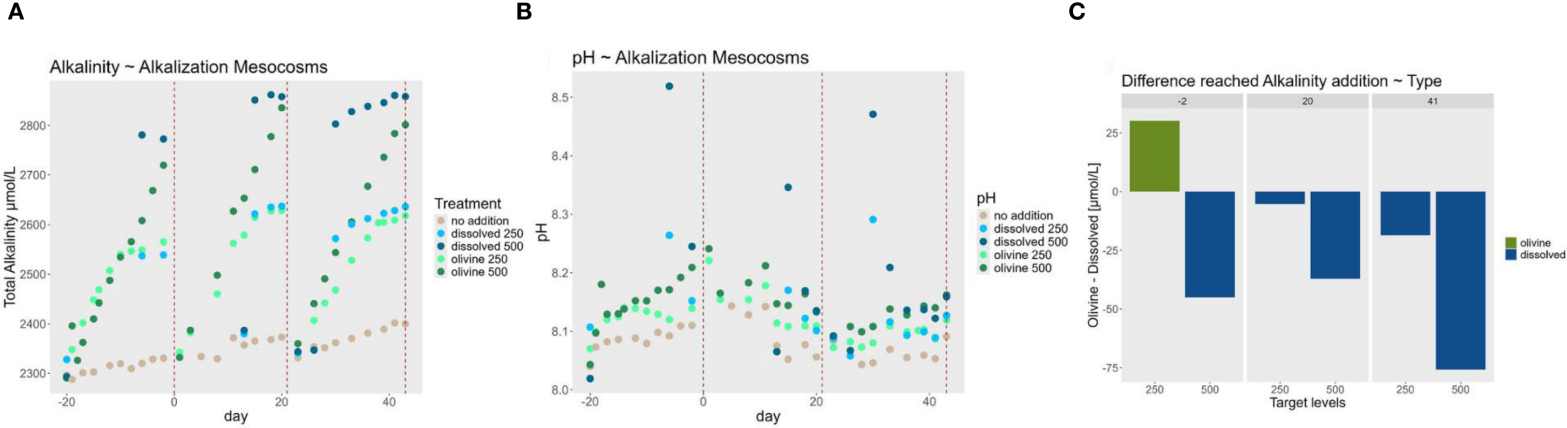
Alkalinity trends **(A)** Alkalinity concentrations in the alkalization mesocosms during each alkalization phase of the experiment. The red dotted lines represent the timepoints in the experiment, when the water from the alkalization mesocosms was pumped to the exposure mesocosms. Measurements are technical triplicates with an average standard deviation of 4,9 µmol·L^-^¹ **(B)** pH measurements during pre-alkalization of the experiment. Points are mean values from technical replicates with average standard deviation of 0.004 values on the pH scale. **(C)** Differences in achieved alkalinity concentrations during the pre-alkalization phase. The y-axis displays the difference in total alkalinity between alkalization types, calculated by subtracting the alkalinity measured in the dissolved enhancement treatment from the corresponding olivine treatment. Positive values (green bars) indicate higher alkalinity levels in the olivine treatment, while negative values (blue bars) indicate higher alkalinity levels in the dissolved treatment. The x-axis shows the targeted alkalinity levels for each treatment tank. Different facets represent the days of the experiment on which measurements were taken before the water was used to expose the oysters.

To evaluate the success of the alkalization the alkalinity of the water in the AMs was monitored during alkalization. For alkalinity and pH measurements, 30 mL of water was titrated using a Metrohm 855 Robotic Titrosampler, following the Gran method (Gran, 1952), with pH 3 as the titration endpoint. The primary goal of these measurements was to monitor the progress of olivine weathering. Alkalinity and pH were measured at least once per week for olivine treatments and before and after each alkalization event in the dissolved NaOH treatments (**Figure 2**). Temperature in the EMs was monitored using a 3630 IDS multi-probe device (Xylem Analytics, Weilheim, Germany) (**Supplementary Figure 2**).

### 2.5 Sampling of water and oyster gills

Sampling of water and gill samples was done in sampling events which took place every three weeks after an alkalization period ended (**Figure 1**). Gill bacterial community samples were taken from EMs, while water samples were taken from AMs. The first sampling event was a baseline sampling of untreated controls. During the baseline event, only one oyster gill and one water sample were collected per treatment. For this, gill samples were taken before alkalized water was transferred to the EMs, and water samples were taken from freshly filled mesocosms prior to the next alkalization cycle. During the second and third sampling events, bacterial community samples were collected in triplicate. The final sampling event included only gill samples, as the experiment was concluding and no further alkalization of water was planned.

For each bacterial community sample, 1 liter of water from the AMs was filtered through 0.22 µm polycarbonate membrane filters (Millipore^®^, MilliporeSigma, Burlington, MA, USA) using vacuum filtration with reusable bottle-top filter units (Nalgene^®^, Thermo Fisher Scientific, Waltham, MA, USA). Filters were placed in 15 mL tubes, flash-frozen in liquid nitrogen, and stored at –20 °C until processing.

To sample the bacterial community from oyster gills, oysters were opened with a shucking knife, and gills were aseptically removed using sterilized scissors. All tools were cleaned and disinfected between each individua. Gill samples were placed in 2 mL cryovials, flash-frozen in liquid nitrogen, and stored at –80 °C until further processing.

### 2.6 DNA extraction

DNA was extracted from water samples using the PowerWater^®^ DNA Isolation Kit (Qiagen, Hilden, Germany), with minor modifications. Modifications included a 10-minute incubation step between steps 5 and 6 to improve yield from hard-to-lyse cells, and elution with 80 µL (instead of 100 µL) to increase DNA concentration.

DNA from oyster gills was extracted using the DNeasy Blood & Tissue Kit^®^ (Qiagen, Hilden, Germany). For DNA extraction, bacterial cells needed to be suspended and separated from the oyster gills. For this, a stock solution of MilliQ water with sodium chloride (NaCl) was prepared at the same salinity as the mesocosm water (32 PSU), to prevent bacterial cell lysis during separation. One gram of 0.1 mm zirconium beads was added to the cryovial containing the gill tissue. Samples were vortexed for 20 minutes at maximum speed using a horizontal adaptor to disrupt the gill tissue. Afterwards, samples were centrifuged at 1,500 × g for 3 minutes. The supernatant, containing the suspended bacterial cells, was transferred to a clean tube. The original tube with oyster tissue was discarded. The supernatant was then centrifuged at 20,000 × g for 10 minutes to pellet the bacterial cells; the supernatant was discarded. The pellet was resuspended in 180 µL ATL buffer and 20 µL Proteinase K (provided by the kit) and incubated at 56 °C until fully dissolved (Rossbach et al., 2019). DNA extraction then followed the manufacturer’s instructions for the DNeasy Blood & Tissue Kit.

### 2.7 PCR and sequencing

Sequencing was performed on an Illumina MiSeq platform using 2 × 300 bp V3 chemistry. Library preparation followed Illumina’s 16S rRNA gene amplicon sequencing protocol (Illumina, 2013). DNA concentrations were quantified using a Quantus fluorometer (Promega Corporation, Madison, WI, USA). 5 ng/µL of DNA from each extract was amplified with PCR using the universal primers 515F-Y (Parada et al., 2016) and 926R (Quince et al., 2011) for bacteria (**Supplementary Table 1**). PCR was validated by visualizing the product with gel electrophoresis before sequencing (**Supplementary Figure 3**). A two-step PCR was used, with the second reaction attaching Illumina indices and adapters according to the manufacturer’s protocol. Amplicons were purified using Agencourt AMPure XP magnetic beads (Beckman Coulter, Inc., IN, USA). After purification, amplicons were re-quantified and pooled at a final concentration of 1 nM per sample, with a 1 nM PhiX control added.

The raw sequencing data was published in the NCBI Sequence Read Archive (SRA) under BioProject accession number: PRJNA1249287.

### 2.8 Bioinformatical processing/ASV generation

Reads were demultiplexed using QIIME 2 (Version 2024.2) (Bolyen et al., 2019) on Linux ubuntu. Primers and indices were removed using cutadapt. Further processing and ASV generation were performed in R (R Core Team, 2020) using DADA2 (Callahan et al., 2016). Reads were quality-filtered by trimming low-quality bases until all positions had a Phred quality score of ≥30 (Prodan et al., 2020). Forward and reverse reads were trimmed to lengths of 250 bp and 200 bp, respectively. Afterwards forward and reverse reads were merged with an overlap of 12 bp. Dereplicated reads were processed with the dada() function, generating amplicon sequence variants (ASVs) (Eren et al., 2013) using a run-specific error model Chimeric sequences were removed, and taxonomic classification was performed using the IdTaxa() function (Murali et al., 2018) in the DECIPHER package (Wright, 2016), with the SILVA database 138 SSU (Quast et al., 2012). Non-bacterial ASVs were excluded from the analysis. One water sample (Sample.ID=82) was removed from the analysis because it had only 40 ASVs.

### 2.9 Testing of treatment effects on gill and water bacterial communities

Treatment effects were analyzed separately for water and gill bacterial communities. One subset included bacterial communities from water samples and the other included bacterial communities from gill samples. Analysis of gill bacterial communities was restricted to samples collected during the final sampling event.

#### 2.9.1 Analysis of the water samples

For the analysis of bacterial communities in water samples, beta diversity was assessed using a Bray–Curtis dissimilarity matrix on rarefied, square root-transformed count data (Bray and Curtis, 1957). This distance matrix was used for Principal Coordinates Analysis (PCoA) (Gower, 1966). Separate PCoAs were generated to assess whether alkalization type or alkalinity concentration was the primary driver of beta diversity differences. Statistical differences were tested using PERMANOVA, implemented via the adonis() and pairwise.adonis() functions (Martinez Arbizu, 2020). Tested variables included sampling date, alkalinity enhancement type (olivine versus dissolved), and treatment concentration. *Post hoc* comparisons between group pairs were conducted using Bonferroni correction to control for type I error (Bland and Altman, 1995). Baseline samples were excluded from all statistical analyses, as they were untreated. The DESeq2 package (Love et al., 2014) was used to identify ASVs with significantly different abundances between alkalization types. These ASVs were used to generate stacked bar plots illustrating differences in bacterial community composition between alkalization types. Alpha diversity metrics (richness and Shannon index) were calculated using the Phyloseq package (McMurdie and Holmes, 2013) and the Vegan package in R (Oksanen et al., 2013). The Kruskal-Wallis test (Kruskal and Wallis, 1952) was applied to assess effects of alkalization type on richness and evenness. *Post hoc* comparisons were performed using pairwise Wilcoxon rank-sum tests (Wilcoxon, 1945) with Bonferroni correction for multiple testing.

#### 2.9.2 Analysis of the gill samples

PCoA, PERMANOVA and identification of ASVs with significantly different abundances with DESeq2 were also performed on the gill community samples, following the same workflow used for water samples. A heat map was generated using the pheatmap package (Kolde and Kolde, 2015) to visualize treatment-specific differences in community composition among DESeq2-identified ASVs. Alpha diversity metrics and associated statistical tests were applied to gill samples using the same approach as for water samples. The only difference was that gill samples were filtered to include a minimum of 250 total counts for alpha diversity analysis.

#### 2.9.3 Overlap between water and gill microbiome

We analyzed how the water bacterial community in each mesocosm influenced the gill-associated community in oysters. Subsets from the final water and gill sampling events were generated for each treatment. The ASVs of these subsets were further ordered into groups specifying where these ASVs were found. The ‘Gill’ and ‘Water’ groups contained ASVs unique to each sample type, while the ‘Overlap’ group included ASVs found in both. Presence–absence data for each ASV were used to generate Venn diagrams with the VennDiagram package (Chen and Boutros, 2011). To quantify how the water community influences the gill community, percentages were calculated, specifying how big the overlapping fraction is from the entire number of ASVs in the gills. “Stacked bar plots were used to visualize the bacterial communities in the ‘Gill’, ‘Water’, and ‘Overlap’ groups based on unrarefied count data, since normalization would have disproportionately reduced diversity in the water samples.

All R code and accompanying metadata required to reproduce the analyses and figures in this study are available at the following GitHub repository: https://github.com/Dom-Antoni/Oyster.

## 3 Results

### 3.1 Reached alkalinity levels

Monitoring of alkalinity levels during the alkalization phase revealed that the target concentrations of 250 µmol·L^-^¹ and 500 µmol·L^-^¹ were not comparably achieved between the alkalization types (**Figure 2**). On average, the measured values deviated by 36 µmol·L^-^¹ from the intended targets. The 500 µmol·L^-^¹ concentration was particularly difficult to achieve with olivine, showing an average shortfall of 86 µmol·L^-^¹ relative to the target. In contrast, the dissolved alkalinity treatments more consistently reached the target levels, falling short by an average of only 23 µmol·L^-^¹. In all but the first exposure period of the 250 µmol·L^-^¹ treatments, measured alkalinity was consistently higher in the dissolved treatments compared to the corresponding olivine treatments. The greatest divergence occurred during the final exposure period of the 500 µmol·L^-^¹ treatments, where the dissolved treatment added 75 µmol·L^-^¹ more alkalinity than the olivine treatment.

### 3.2 Sequencing reads and ASV yields

The number of raw sequenced reads varied significantly between gill and water samples. Gill samples had an average of 56,878 reads, while water samples yielded nearly three times as many, with an average of 142,832 reads. On average, the most abundant read in gill samples accounted for 75.67% of total reads. NCBI BLAST analysis identified this read as matching the small ribosomal subunit of *Ostrea edulis*, with 100% coverage (accession no.: XR_008798941).

ASV yields from gill samples were also substantially lower than those from water samples. The median ASV count in gill samples was 234, compared to 81,548 in water samples, representing a difference of two orders of magnitude. ASV yield in gill samples also varied greatly, ranging from as few as 3 to over 3,000 ASVs. A trend was observed of increasing ASV yield over time in the gill samples. At the final sampling event, all gill samples yielded at least 250 ASVs.

### 3.3 Bacterial community composition in water samples

**Figure 3:**
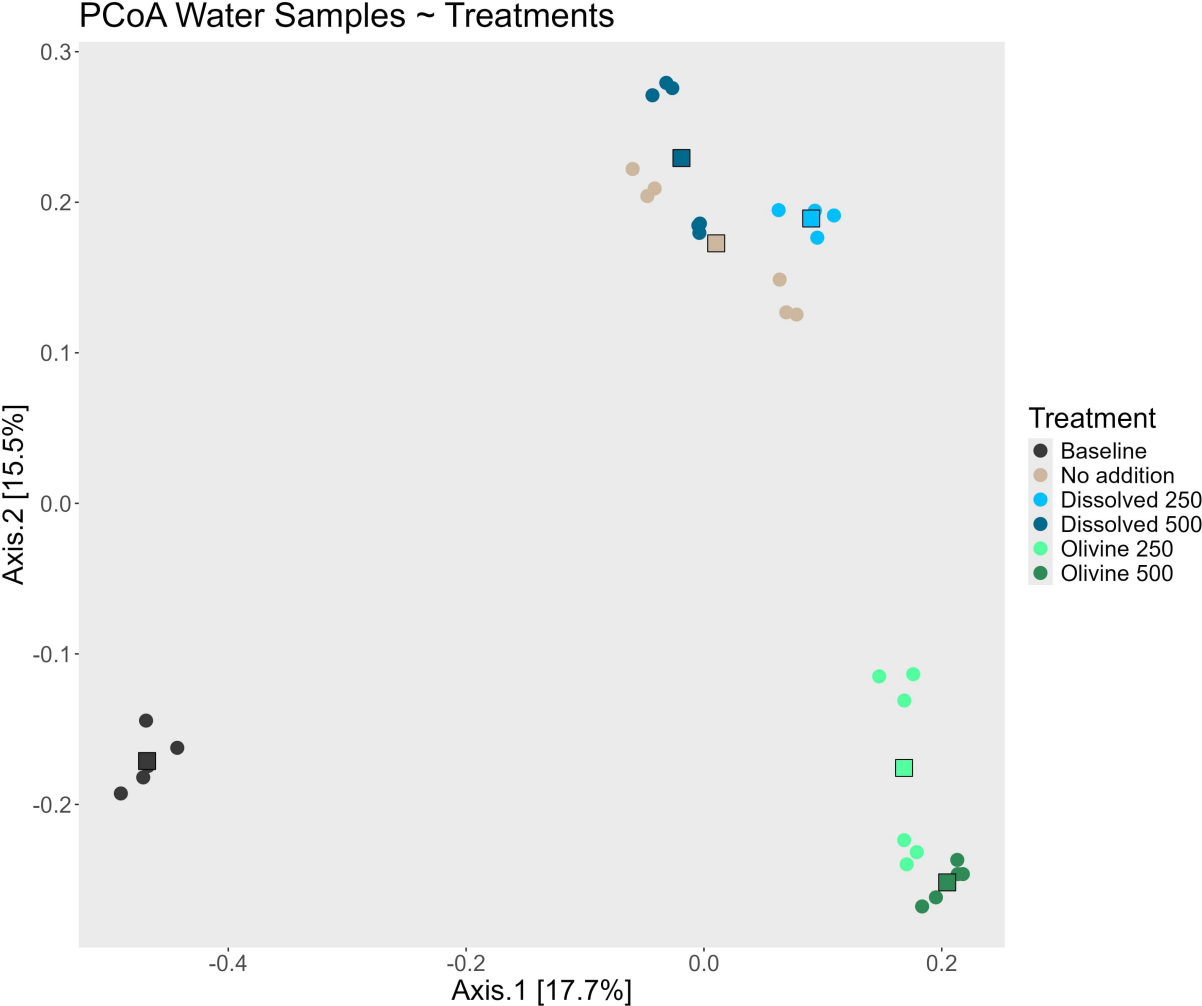
PCoA of the water samples taken during the experiment colored by the different treatments. The round points represent one water sample, while the squares represent the centroid of each treatment. The “Baseline” treatment consists of sample taken from each mesocosm prior to any treatment addition, while the “No addition” treatment are samples taken from the same mesocosm witch never had alkalinity added to but ran along as a control for the mesocosm setup over the course of the experiment.

**Figure 4:**
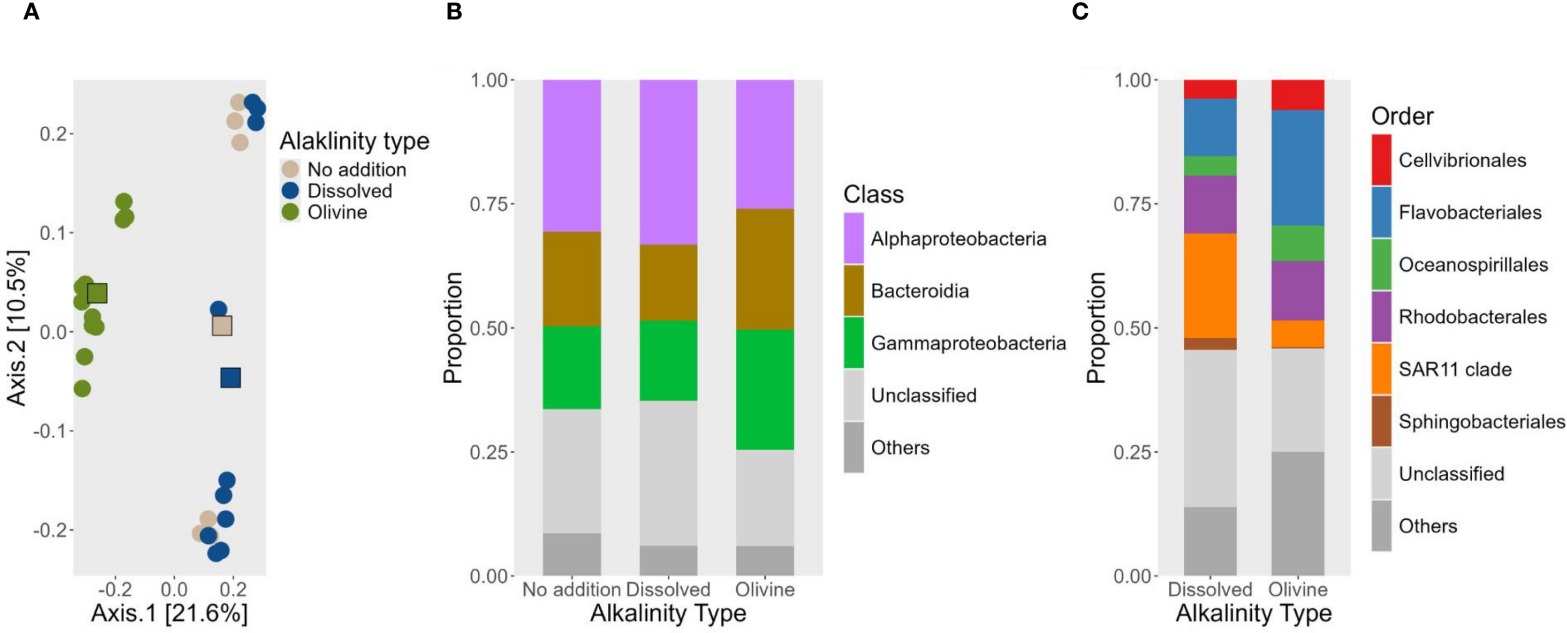
**(A)** PCoA to visualize the statistical tests on how the alkalization type is affecting the water community samples. This PCoA is not including the Baseline samples, as they are untreated samples and would bias the statistical analysis and the beta diversity analysis. **(B)** Stacked bar plots showing the bacterial community composition at the taxonomic class level between the different alkalization types. A filter is applied where taxonomic classes with less than a five percent share of the community composition are lumped into the “Others” fraction. For the generation of this plot only ASVs are used which were found to be significant by the DESeq2 package. **(C)** Stacked bar plot at the taxonomic order level between the dissolved OAE alkalization type and the olivine alkalization Type. A filter is applied where taxonomic orders with less than an eight percent share of the community composition are lumped into the “Others” fraction. For the generation of this plot only ASVs are used which were found to be significant by the DESeq2 package.

The two main axes of the Principal Coordinates Analysis (PCoA) of all water samples captured 32.1% of the total variability. Distinct cluster formation corresponding to the applied treatments is visible in the PCoA (**Figure 3**). Samples from the olivine treatments formed a separate cluster from those of the other treatments. Samples from the dissolved alkalinity treatments formed their own cluster, although their centroids were close to that of the ‘no addition’ control. Within the clusters formed by the two alkalization types, samples associated with different concentrations formed distinct sub-clusters. However, the primary driver of beta diversity in the water bacterial communities was the alkalization type. A separate PCoA including only the treated samples (**Figure 4A**) visualized this distinction more clearly. Microbial communities in olivine-treated waters formed a distinct cluster, with its centroid markedly separated from those of the dissolved and untreated samples. Using PERMANOVA, we tested the effects of sampling date, alkalinity enhancement type, and treatment concentration on centroid position. All parameters had statistically significant effects (p < 0.05; **Table 1**). *Post-hoc* pairwise comparisons confirmed significant differences for both sampling date and alkalinity enhancement type. However, treatment concentration effects were only statistically significant in comparisons involving at least one olivine treatment (**Table 2**).

**Table 1:** PERMANOVA test for statistical effects of different parameters on the different microbiome sample types.

Water			Gills		
Parameter	p-value	Sig.	Parameter	p-value	Sig.
Date	0.006	*			
Type	0.001	*	Type	0.047	*
Treat.	0.001	*	Treat.	0.001	*

P-values from pairwise comparisons are Bonferroni-adjusted. Rows with a star in the “Sig.” Column highlight row with (p < 0.05).

The symbol "*" means that the p-value in this row is significant and thus smaller than 0.05.

**Table 2:** Pairwise comparisons between parameter groups from **Table 1**, with Bonferroni-adjusted p-values. Significance was set at p-adjust < 0.05.

Water				Gills				
Parameter	Pairs	p-value	p-adjust	Sig.	Pairs	p-value	p-adjust	Sig.
Date								
	28.5.2024 ~ 18.6.2024	0.003	0.003	*				
Type								
	no add. ~ oli.	< 0.001	0.003	*	no add.~oli.	0.350	1	
	no add. ~ dis.	0.008	0.024	*	no add.~dis.	0.074	0.222	
	Oli. ~ dis.	< 0.001	0.003	*	Oli.~dis.	0.118	0.354	
Treat.								
	no add. ~ oli. 250	0.001	0.01	*	no add.~oli.250	0.7	1	
	no add. ~ oli. 500	0.003	0.03	*	no add.~oli.500	0.1	1	
	no add. ~ dis. 250	0.007	0.07		no add.~dis.250	0.1	1	
	no add. ~ dis. 500	0.023	0.23		no add.~dis.500	0.1	1	
	oli. 250 ~ oli. 500	0.002	0.02	*	oli.250~oli.500	0.1	1	
	oli. 250 ~ dis. 250	0.007	0.07		oli.250~dis.250	0.1	1	
	oli. 250 ~ dis. 500	0.005	0.05		oli.250~dis.500	0.1	1	
	oli.500 ~ dis.250	0.008	0.08		oli.500~dis.250	0.1	1	
	oli. 500 ~ dis. 500	0.002	0.02	*	oli.500~dis.500	0.1	1	
	dis. 250 ~ dis. 500	0.021	0.21		dis.250~dis.500	0.1	1	

P-values from pairwise comparisons are Bonferroni-adjusted. Rows with a star in the “Sig.” Column highlight row with (p < 0.05).

The symbol "*" means that the p-value in this row is significant and thus smaller than 0.05.

DESeq2 analysis revealed no significantly different ASVs between the dissolved enhancement type and the ‘no addition’ control. In contrast, the olivine enhancement type showed 48 significantly different ASVs compared to the ‘no addition’ control. The largest number of differentially abundant ASVs was observed between the olivine and dissolved alkalinity treatments, with 101 ASVs. These ASVs, which differed significantly among treatments, were visualized using stacked bar plots to highlight shifts in community composition at the bacterial class level (**Figure 4B**). Dissolved alkalinity treatments showed an increase in Alphaproteobacteria and a decrease in Bacteroidia. Olivine treatments showed an increase in Gammaproteobacteria, accompanied by a decrease in Alphaproteobacteria. However, olivine-enhanced treatments exhibited a greater increase in Bacteroidia compared to both dissolved alkalinity and ‘no addition’ controls. To further highlight differences between the two OAE materials, significantly different ASVs were also visualized at the order level (**Figure 4C**). Olivine-treated samples had a higher fraction of Cellvibrionales and Flavobacteriales, while the SAR11 clade was more abundant in dissolved alkalinity treatments. The relative abundance of the Rhodobacterales class did not change markedly. However, the figure includes only those ASVs that showed significant differences between treatments. This suggests that ASVs within the Rhodobacterales were differentially affected by the two alkalization types.

Samples from olivine-treated waters exhibited reduced bacterial alpha diversity. The median richness of olivine-treated samples was 884. In comparison, the dissolved alkalinity treatment and the ‘no addition’ control had median richness values of 1,081 and 1,096, respectively. The Shannon index was also lower in olivine-treated samples (6.37) compared to dissolved alkalinity-treated samples (6.61) and ‘no addition’ controls (6.63). Statistical analysis indicated that both richness and evenness were significantly affected by the alkalization type (p < 0.05). Pairwise *post hoc* tests showed that significant differences occurred only in the olivine-treated water samples (**Table 3**).

**Table 3:** Kruskal-Wallis and pairwise Wilcoxon test results for alpha diversity (Richness and Shannon Index) in water and gill microbiomes.

Diversity metric	Water			Gills		
	Richness	Shannon	Sig.	Richness	Shannon	Sig.
Statistical test	0.0072	0.0085	*	0.5414	0.3216	
Pairwise Comparisons						
Baseline vs. No addition	1	1		1	1	
Baseline vs. Dissolved	1	1		1	0.53	
Baseline vs. Olivine	0.31	0.31		0.27	1	
No addition vs. Dissolved	1	1		1	1	
No addition vs. Olivine	0.029	0.029	*	1	1	
Dissolved vs. Olivine	0.029	0.048	*	1	1	

P-values from pairwise comparisons are Bonferroni-adjusted. Rows with a star in the “Sig.” Column highlight row with (p < 0.05).

The symbol "*" means that the p-value in this row is significant and thus smaller than 0.05.

### 3.4 Oyster microbiome responses

**Figure 5:**
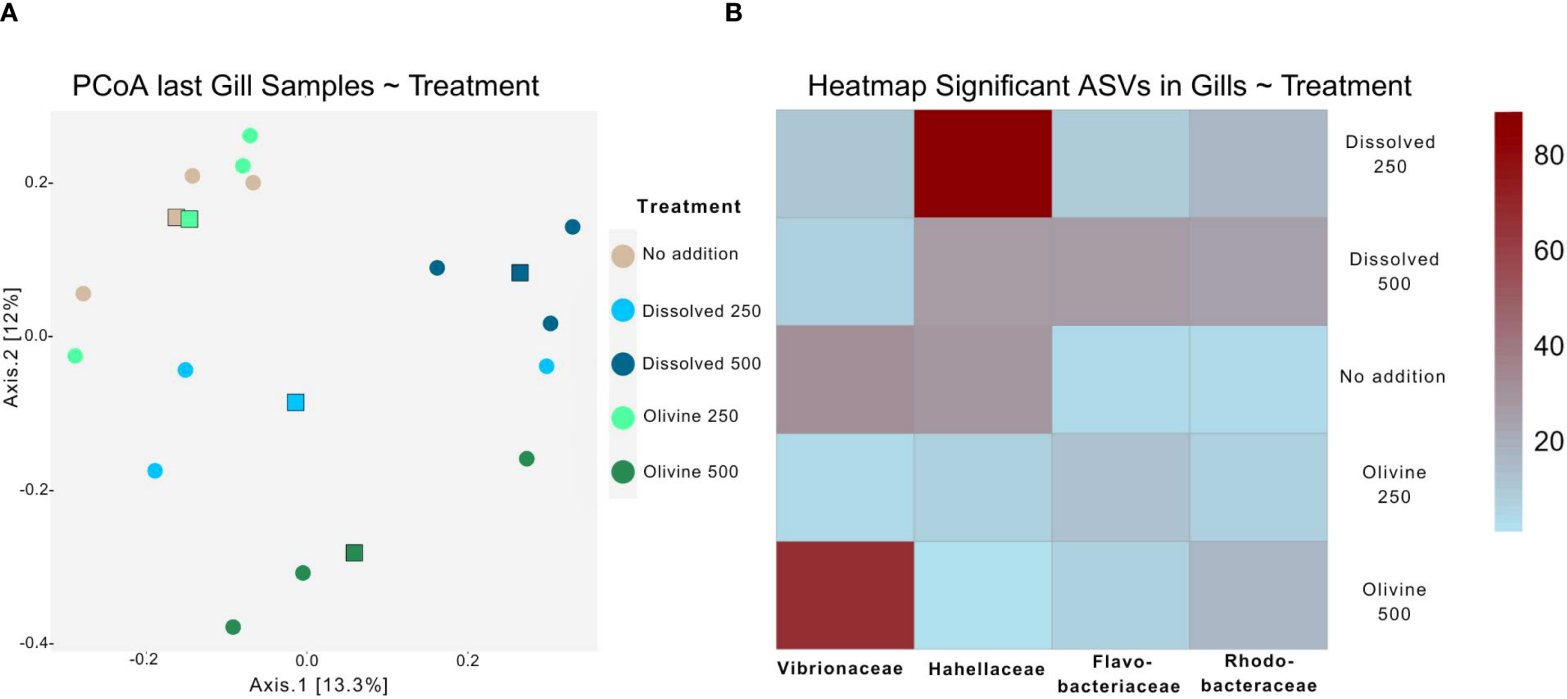
**(A)** PCoA visualizing the gill microbiome of the last sampling event of the experiment color coded by the treatments used in the experiment. The round points depict community samples while the squares represent the centroid of the treatments. **(B)** Heatmap depicting the distribution of ASVs found to be significantly different among treatments with DESeq2 in gill samples from the last sampling time point. The Abundance in the legend are Absolut values after normalization to the same sequencing depth of 250 counts between samples. The Taxonomic group shown is the family level.

The two main axes of the PCoA of the gill microbiome samples explained 25.3% of the variability (**Figure 5A**). This was 6.8% lower than the variance explained by the PCoA of the water samples. Cluster formation and centroid separation among treatment groups were less distinct than in the water sample PCoA. Oyster gill microbiomes were more influenced by alkalinity concentration than by enhancement type. Samples from the 500 µmol·L^-^¹ treatments clustered further from the control than those from the 250 µmol·L^-^¹ treatments. However, enhancement type also influenced the oyster gill microbiome, as the two 500 µmol·L^-^¹ treatments were more dissimilar to each other and diverged in different directions relative to the ‘no addition’ control in the PCoA. PERMANOVA testing of the gill samples showed significant effects of both alkalinity enhancement type and underlying treatment concentration (p < 0.05; **Table 1**). However, subsequent *post hoc* tests did not reveal statistically significant differences between any treatment pairs.

Four ASVs showed significantly different occurrences between enhancement types in the gill samples. Their distribution across treatments was shown in a heatmap (**Figure 5B**). The 500 µmol·L^-^¹ olivine treatment had a higher abundance of an ASV associated with Vibrionaceae, whereas the corresponding dissolved treatment had higher abundances of Flavobacteriaceae and Rhodobacteraceae. The 250 µmol·L^-^¹ dissolved treatment showed increased abundance of Hahellaceae, which were nearly absent in the olivine treatments.

**Figure 6:**
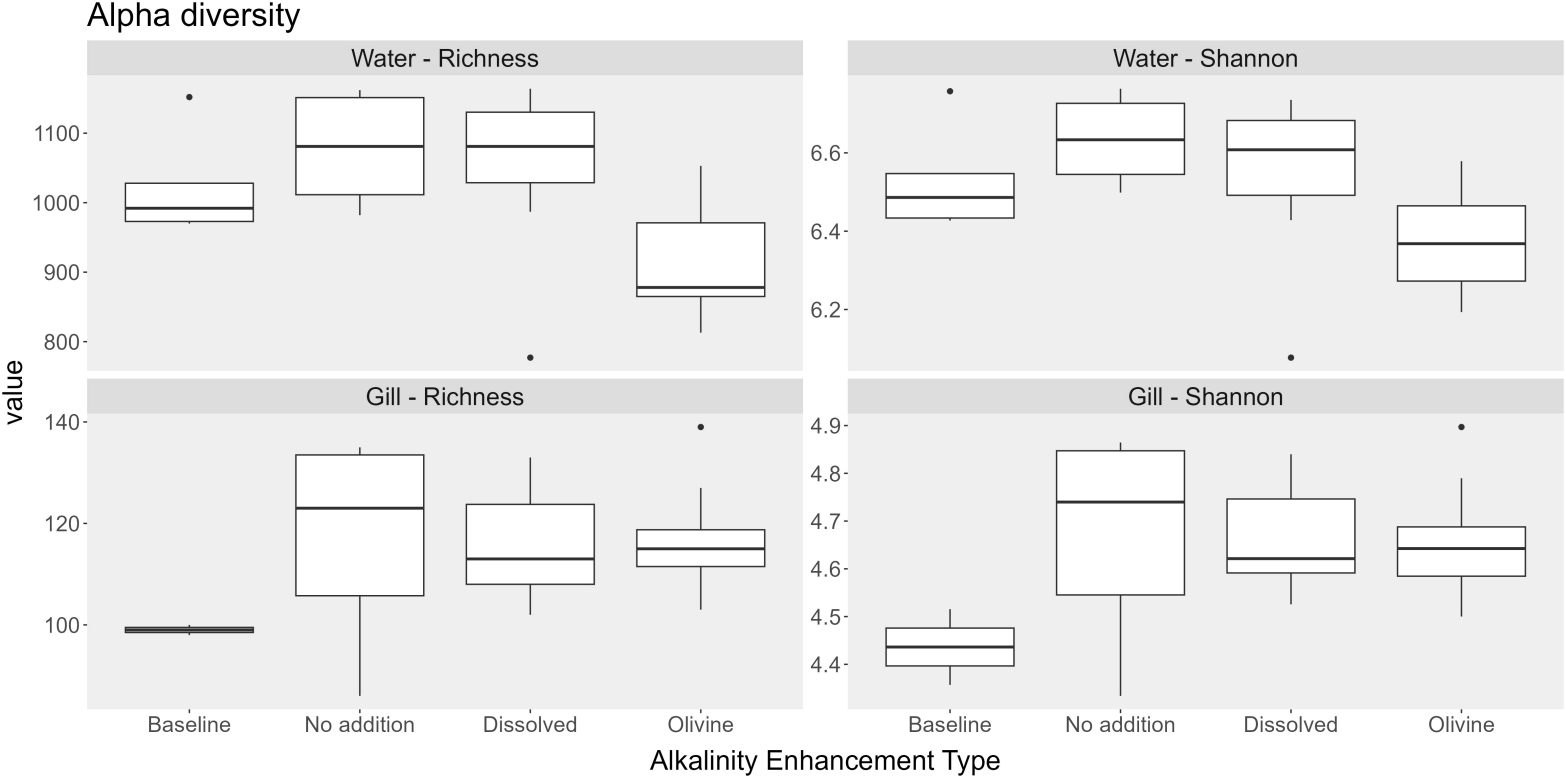
Boxplots showing alpha diversity (Richness and Shannon index) in water and gill microbiomes across the different alkalization types. Types include Baseline (pre-treatment samples), No addition (mesocosms without alkalinity addition), Dissolved (addition of NaOH), Olivine alkalinity (addition of weathered Olivine). Sample sizes per treatment are indicated as *n* (water; gill): Baseline (*n* = 5; 4), No addition (*n* = 6; 12), Dissolved (*n* = 11; 16), Olivine (*n* = 11; 16). The numbers represent the number of water and gill samples, respectively.

The alpha diversity of the bacterial communities in the gill samples did not differ significantly among alkalization types (**Figure 6**). Median richness ranged from 118 in the baseline sample to 198 in the ‘no addition’ controls. The baseline samples had the lowest Shannon index (3.76), while the ‘no addition’ controls had the highest (4.92). Statistical tests showed no significant differences in alpha diversity metrics between the alkalization types (**Table 3**).

### 3.5 Overlapping taxa between water and gill

**Figure 7:**
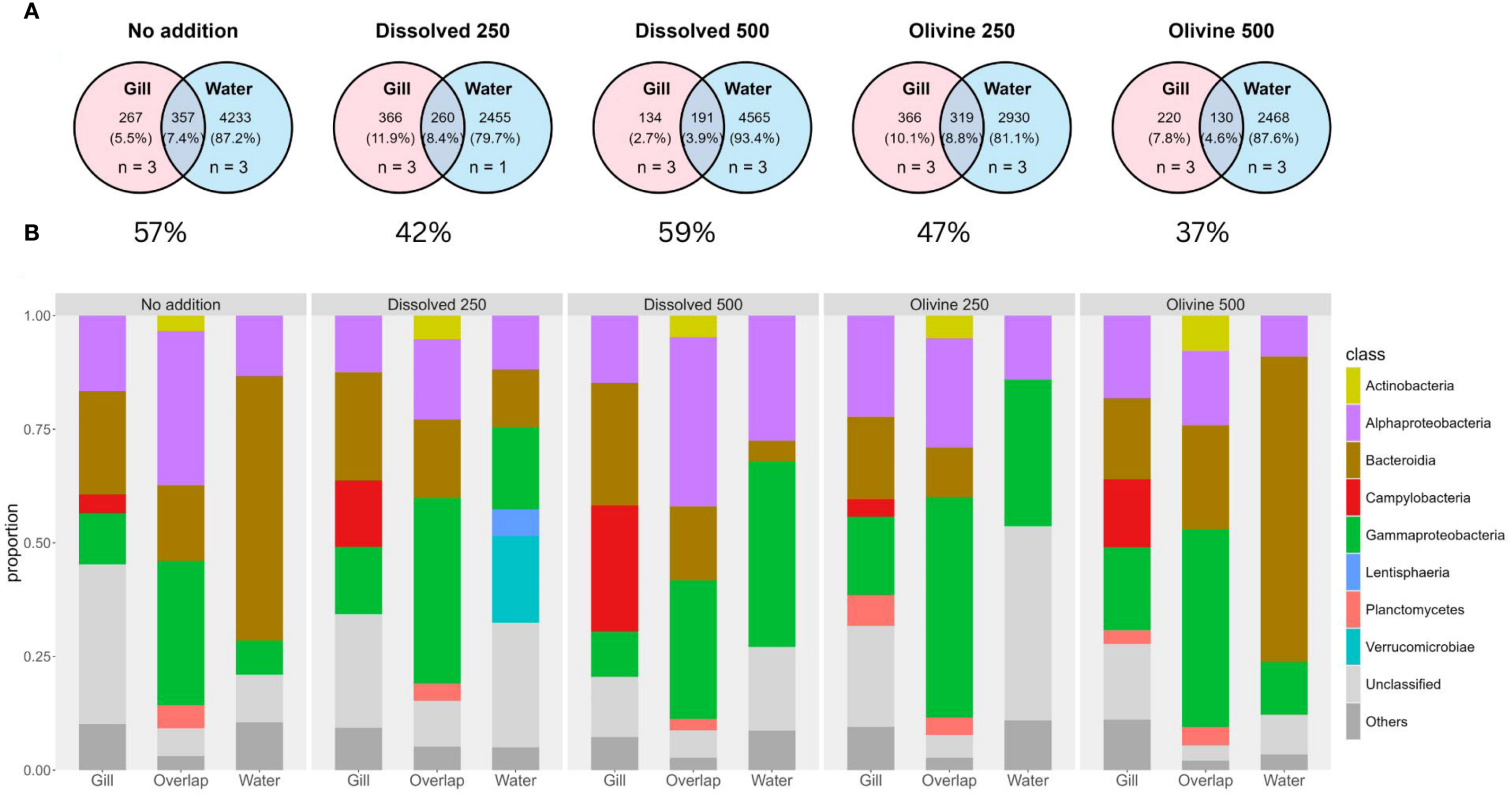
**(A)** Venn diagrams showing the number of unique and overlapping ASVs in treated water samples (used for oyster exposure) and oyster gill samples taken three weeks post-exposure. Displayed percentages show the size fraction which the “Overlap” group is from all ASVs found on the gill samples. Data is based on unrarefied presence/absence tables. The number of replicate samples per group is indicated by “n”. **(B)** Stacked bar plots showing the bacterial community composition of the samples in panel **(A)** ASVs are grouped into three categories per treatment: unique to gill samples, unique to water samples, and shared between both (“Overlap”). Colors indicate bacterial taxonomic classes and “others” is a grouping of classes with less than 5% share of the community composition.

Venn diagrams summarize the number of amplicon sequence variants (ASVs) shared between oyster gill and water samples per treatment (**Figure 7A**). Across all conditions, the majority of ASVs were unique to water samples (79.7%–93.4%), while a smaller fraction was specific to gills (2.7%–11.9%). The overlap between gill and water ASVs ranged from 3.9% to 8.8%. Each Venn diagram in **Figure 7A** indicates the proportion of overlapping ASVs relative to all ASVs found in gill samples. The fraction of overlapping ASVs is at least 37% in each treatment of all ASVs found in gill samples.

The bacterial communities in the ‘Gill’, ‘Water’, and ‘Overlap’ groups showed distinct patterns that did not appear treatment-specific (**Figure 7B**). ASVs unique to the water samples showed treatment-specific variation, particularly in the relative abundance of Bacteroidia and Gammaproteobacteria. The ‘no addition’ control and the 500 µmol·L^-^¹ olivine treatment were both dominated by Bacteroidia, while the other treatments had higher proportions of Gammaproteobacteria. The 250 µmol·L^-^¹ dissolved treatment was the only one that showed visible fractions of Verrucomicrobiae and Lentisphaeria. ASVs unique to the gill samples revealed a stable bacterial community across treatments, dominated by Alphaproteobacteria and Bacteroidia, with varying fractions of Campylobacteria and Gammaproteobacteria. The fraction of Campylobacteria in the gills increased with higher alkalinity treatments. The ‘Overlap’ group also had stable community compositions across treatments. The dominant taxa in the ‘Overlap’ group were Gammaproteobacteria, Bacteroidia, and Alphaproteobacteria, with only slight variation across treatments.

### 3.6 Oyster mortality rate

After the exposure period ended, oyster mortality rates varied across treatments. The lowest mortality rate (20%) occurred in the control treatment without added alkalinity. Mortality rates increased with higher alkalinity concentrations in both enhancement types. In the 250 µmol·L^-^¹ treatments, mortality was 23% in the olivine group and 30% in the dissolved group. In the 500 µmol·L^-^¹ treatments, mortality rose to 38% in the olivine group and 33% in the dissolved group.

## 4 Discussion

### 4.1 Sequencing success

The number of raw sequences, and consequently the ASV yield, was substantially lower in gill samples compared to water samples. The MiSeq sequencing platform is known to produce variable sequencing depths across samples (Wen et al., 2017). However, the extent of variability observed in this sequencing run was unusual, spanning over four orders of magnitude. This discrepancy was likely due to the choice of primers used for amplifying the 16S rRNA gene. The high fraction of sequences associated with the ribosomal subunit of *Ostrea edulis* indicates that the chosen primers were not specific enough to exclude host contamination. Other studies reporting higher ASV yields in oyster microbiome analyses used a V4 primer pair (515f and 806r) (Pimentel et al., 2021; Singh et al., 2022; Unzueta-Martínez et al., 2022). We used a V4–V5 primer pair, opting for a different reverse primer (Parada et al., 2016). This pair performs well in marine waters (Gilbert et al., 2018) and was selected to minimize primer bias. However, this pair appears to be insufficiently specific for host microbiome studies, particularly when comparisons to surrounding water microbiomes are intended. In a study that compared the bacterial communities in oyster gills and surrounding water, the same V4–V5 primer pair was used (Diner et al., 2023). Similar alpha diversity metrics were reported in that study, suggesting that ASV yields are representative and reproducible with these primers.

Inspection of PCR validation gels revealed that amplicons from gill samples were longer than those from water samples (**Supplementary Figure 3**). Amplicon length influences sequencing success, as shorter reads bind more easily to flow cell sites and are thus more likely to be sequenced. A study evaluating different primer pairs in jellyfish (Barak et al., 2023) reported reduced sequencing depth in samples amplified with the same V4–V5 primers used in our study. The authors note a reduced ASV yield, because the merging of forward and reverse reads was unsuccessful, due to an extended length of the PRC product. We note that the primers used in our study were suboptimal for the analysis of the oyster gill bacterial community. Nonetheless, we observed a trend in which more bacterial ASVs were detected in gill tissue at later sampling time points. This may reflect healthier oysters with lower bacterial loads at the start of the experiment. Stressful conditions for oysters, such as increased temperature, can lead to the accumulation of certain bacteria (Diner et al., 2023). Later in the experiment, the oysters appeared more susceptible to bacterial colonization, as evidenced by the higher abundance of ASVs in the gill samples.

### 4.2 High impact of alkalization with olivine on the bacterial community

We observed a greater impact on bacterial communities in olivine-treated samples compared to those treated with dissolved NaOH. Dissolved alkalization showed no notable differences relative to the control treatment without added alkalinity. This finding aligns with results from a 2023 study by the RETAKE consortium on Helgoland (Antoni et al., 2025). In that study, alkalinity was added using a similar method to our dissolved treatment, but at higher concentrations (up to 1250 µmol·L^-^¹). The authors concluded that temporal changes in the bacterial community were primarily driven by natural succession at the study site. Our findings support this conclusion: the bacterial communities in both the dissolved treatments and the untreated control mesocosms, which reflect natural succession, were highly similar.

Alkalization with olivine led to distinct taxonomic shifts compared to the ‘no addition’ treatment, including increased abundance of Gammaproteobacteria (notably Cellvibrionales), significant changes within the Rhodobacterales class, and a decline in the SAR11 clade. These taxa are indicative of environmentally unfavorable conditions. Increased Gammaproteobacteria abundance is typically associated with nutrient-enriched and polluted waters, such as those near wastewater discharge sites (Buccheri et al., 2019). Rhodobacterales are a well-studied class known for surface colonization and biofilm formation (Michael et al., 2016). The ecological interpretation of shifts in Rhodobacterales abundance remains uncertain, as this diverse group includes both opportunistic pathogens and mutualists. Members of the genus *Loktanella* have been associated with black spot disease in crabs (Bergen et al., 2022). Other members of the Roseobacter clade, however, are used in aquaculture because they produce probiotics that reduce Vibrionaceae abundance (Pintado et al., 2023). While the role of Rhodobacterales remains ambiguous, shifts in their abundance are used as indicators of dysbiosis in aquaculture systems (Unzueta-Martínez et al., 2021). Flavobacteriales were also more abundant in olivine-treated samples. Like Rhodobacterales, Flavobacteriales are known surface colonizers and biofilm formers (Buchan et al., 2014). Cellvibrionales showed a slight increase in olivine-treated waters. This increase raises concern, as Cellvibrionales are associated with eutrophic and environmentally stressed marine systems characterized by reduced microbial diversity (Wong et al., 2024). Furthermore, the genus *Vibrio* includes many pathogenic taxa, such as *V. cholerae* (Vezzulli et al., 2010). Overall, the bacterial communities in olivine-alkalinity-enhanced waters were enriched in taxa associated with biofilm formation, pollution, and nutrient enrichment. This pattern is further supported by the observed decline in the SAR11 clade. Members of the SAR11 clade are mostly unclassified bacteria that typically occur in nutrient-deprived environments such as oceanic gyres (Giovannoni, 2017).

Another notable concern is the reduced diversity observed in olivine-treated samples. This reduction suggests that the previously discussed biofilm-forming and pollution-tolerant taxa are outcompeting other bacteria. Given the reduced diversity and pronounced taxonomic shifts observed in olivine-treated waters compared to dissolved treatments, these differences are likely caused by additional olivine dissolution products rather than alkalinity alone. This is particularly striking considering that olivine treatments generally released less alkalinity than the dissolved treatments. Olivine contains multiple impurities and toxic trace metals like nickel, which can affect microbial communities. Nickel is a known pollutant that can alter microbial diversity and function (Morawska-Płoskonka and Niklińska, 2013). Nickel can affect microorganisms through multiple mechanisms, including inducing oxidative stress and inhibiting enzymatic activity by replacing essential metal cofactors (Macomber and Hausinger, 2011). It is also possible that, beyond its dissolution products, the particulate nature of olivine itself influences microbial community structure. Studies which investigated effects of olivine addition on the environment encountered effects with far lower concentrations of released alkalinity. In the study by Guo et al. (2025) alkalinity addition was below 30 µmol·L^-^¹. These findings support the possibility that olivine as a particle and not the released products from it are causing the observed changes (Ren et al., 2022).

### 4.3 Influence from alkalization on the oyster gill bacterial community

Oyster gill bacterial communities were primarily influenced by alkalinity concentration rather than the alkalization type used. Health is a cofactor for the microbiome of the oyster (Clerissi et al., 2018; Yeh et al., 2020) and we saw an increasing oyster mortality rate with increasing alkalinity concentration. We observed a high mortality rate, with at least 20% of oysters dying in a single treatment. This may be attributable to elevated temperatures during the summer months, as summer mortality events are well-documented in oyster hatcheries and nurseries (King et al., 2019; Noetzel et al., 2025). Temperatures during the final phase of the experiment reached 19–20 °C. This can be a stressor to our oysters who were adapted to temperatures at the bottom of the sea. Nevertheless, higher mortality rates was observed in mesocosms with increased alkalinity, suggesting a negative effect on oyster health. This contrasts with the general belief that calcifying organisms benefit from OAE (Bach et al., 2019; Vicca et al., 2022). One possible explanation for this discrepancy is that OAE may facilitate the proliferation of opportunistic pathogens.

At least 37% of the bacterial ASVs detected in oyster gills were also present in the alkalized waters used for exposure. This overlap is considerably higher than reported in a previous study, which found a maximum of 21% overlap between oyster and surrounding water communities (Liu et al., 2024). The higher overlap in our study may be caused by the extended exposure, as we sampled the water 3 weeks prior to the gills, while Liu et al. (2024) sampled synchronously. Liu et al. (2024) did not detail the oyster processing and it might be that the entire community was assessed, while we focused on the gills, which are the first contact point with the surrounding water. The gill microbiome was influenced strongly by the water and Gammaproteobacteria were particularly dominant overlap between water and gill communities. This is notable because Gammaproteobacteria in olivine-treated waters were enriched in Cellvibrionales. The most abundant ASV in gill samples from the high-concentration olivine treatment belonged to the genus *Vibrio*. Many species from this genus pose significant threats to oysters, often leading to mass mortalities in aquaculture. Notably, *V. aestuarianus*, *V.* sp*lendidus*, *V. coralliilyticus*, and *V. tasmaniensis* are frequently associated with oyster mass mortality events. These pathogens produce virulence factors that enable them to invade, damage, and disrupt oyster physiology. For instance, metalloproteases enzymes like Vam (from *V. aestuarianus*) and Vsm can lyse peptides and interfere with oyster immune cells, impairing their ability to adhere and perform phagocytosis (Vandeputte et al., 2024). Interestingly, a study on ocean acidification in oyster larvae reported a decline in *Vibrio* abundance under acidified conditions (Prado et al., 2016). Such contrasting outcomes between OAE and ocean acidification studies are not uncommon (Antoni et al., 2025). Vibriosis may have contributed to the elevated oyster mortality observed in the 500 µmol·L^-^¹ olivine-exposed mesocosm.

This finding is particularly important in the context of assessing the ecological risks of OAE. The observation that olivine-amended waters may promote *Vibrio* proliferation and potentially contribute to vibriosis in host organisms is novel. It is highly relevant to the ecotoxicological assessment of olivine as an OAE application strategy. Importantly, dissolved alkalinity treatments appeared less ecologically disruptive than olivine, even for the oyster microbiome, despite achieving higher alkalinity levels in the experiment. These results suggest that NaOH-based OAE carries a lower ecological risk than olivine.

### 4.4 Outlook and future directions

Biotic responses and environmental impacts differ substantially between OAE scenarios. In the case of the experiment presented here, some impacts were not fully explainable by the concentration of the alkalinity released. Further targeted research is required to systematically compare a wider range of OAE strategies and to identify those that maximize CO_2_ sequestration while minimizing ecological risks, as called for by authors of Dupont and Metian (2023). Future investigations could also explore alternative enhancement approaches such as electrochemical alkalinity generation or the use of other alkaline materials like basalt and quicklime.

For studies involving similar setups like ours, with distinct sample types such as host tissues and seawater, we recommend the consideration of employing different primers for the microbiome analyses between host organism and water. While this introduces primer-specific bias, it may prevent underrepresentation of specific microbial communities due to suboptimal amplification by universal primers in a specific sample type. Additionally, expanding the range of investigated host organisms is suggested. Macroalgae are promising candidates in coastal ecosystems, serving not only as key carbon reservoirs but also as nature-based NETs (Pessarrodona et al., 2023). Their inclusion would provide valuable contrast to calcifying model organisms and would broaden the ecological relevance of OAE assessments.

As our understanding of biological impacts across OAE strategies improves, ecotoxicological effects may become increasingly predictable, enabling environmentally informed application scenarios to be tested in field studies. The transition from controlled mesocosm experiments to field-scale trials represents a necessary next step in evaluating OAE as a viable NET (Riebesell et al., 2023). Each additional study contributes to building a knowledge base that may support the future deployment of OAE in governmental or private climate initiatives aimed at mitigating the effects of global warming and reducing atmospheric CO_2_ concentrations.

## 5 Conclusion

Ocean alkalinity enhancement using olivine poses greater ecotoxicological risks than alkalization with dissolved NaOH. Whereas bacterial communities in the NaOH treatments resembled those of untreated controls, olivine-treated waters were enriched in pollution-resistant and biofilm-forming taxa, such as Gammaproteobacteria and Flavobacteriales. This bacterial dysbiosis may influence the microbiomes of marine animals, such as oysters. Opportunistic pathogens may proliferate in host organisms, as evidenced by the increased abundance of *Vibrio* taxa in oysters and the associated rise in oyster mortality. These findings underscore the importance of careful material selection for OAE strategies. Despite releasing less alkalinity, olivine posed a greater ecotoxicological risk than NaOH. This study provides initial ecotoxicological insights to inform the evaluation of different OAE materials. These findings should be considered in future discussions on the implementation of marine carbon dioxide removal strategies to minimize unintended ecological impacts.

## Data Availability

The raw sequencing data generated in this experiment is uploaded at the NCBI as a BioProject repository with the accession number PRJNA1249287. The R Code used to generate the ASVs and the code used to generate plots and the further aspects for the ecological analysis are publicly available on GitHub under the following link: https://github.com/Dom-Antoni/Oyster.
