# Supplementary material for: Olivine and dissolved alkalinity trigger different bacterial community shifts in water and oyster gills: insights from a mesocosm experiment
Source: Front Microbiomes. 2025 Sep 26;4:1659695. doi: 10.3389/frmbi.2025.1659695 (PMC12993672; doi:10.3389/frmbi.2025.1659695)
Supplement: Supplementary file 1 [file Supplementaryfile1.docx]

Supplementary Material


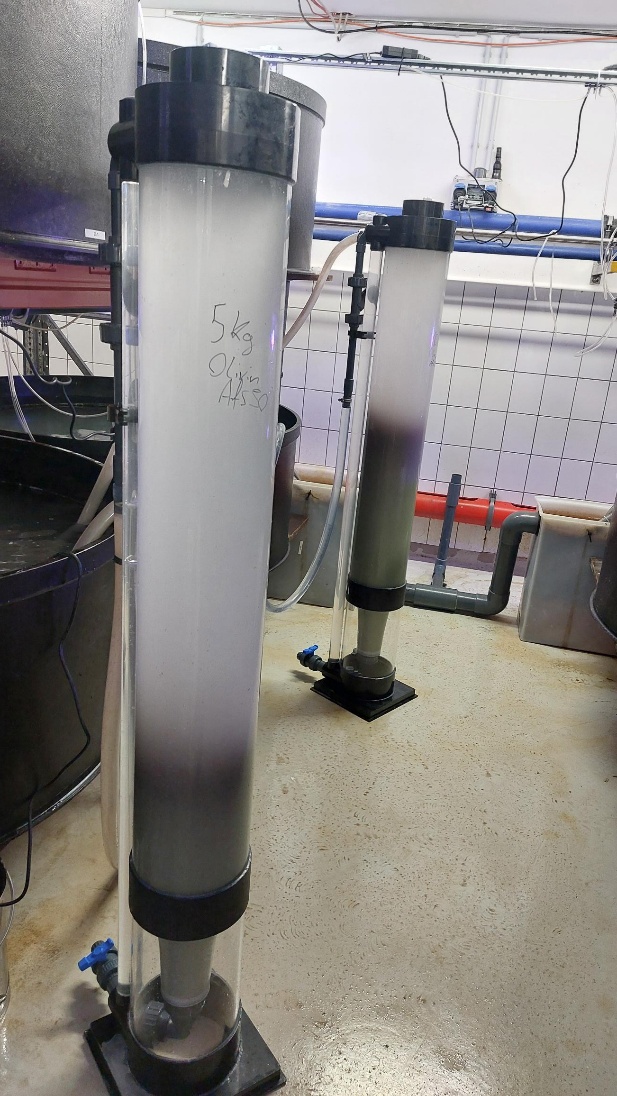


**Supplement Figure 1:** Setup of the olivine treatments. The alkalization mesocosms (AM) are the black tanks in the lower shelfs. The fluidized bed filters (FBF) are the columns standing in the middle of the hall way. Alkalinity in enhanced by pumping the water constantly through the FBF which has olivine AFS 50 inside of it.


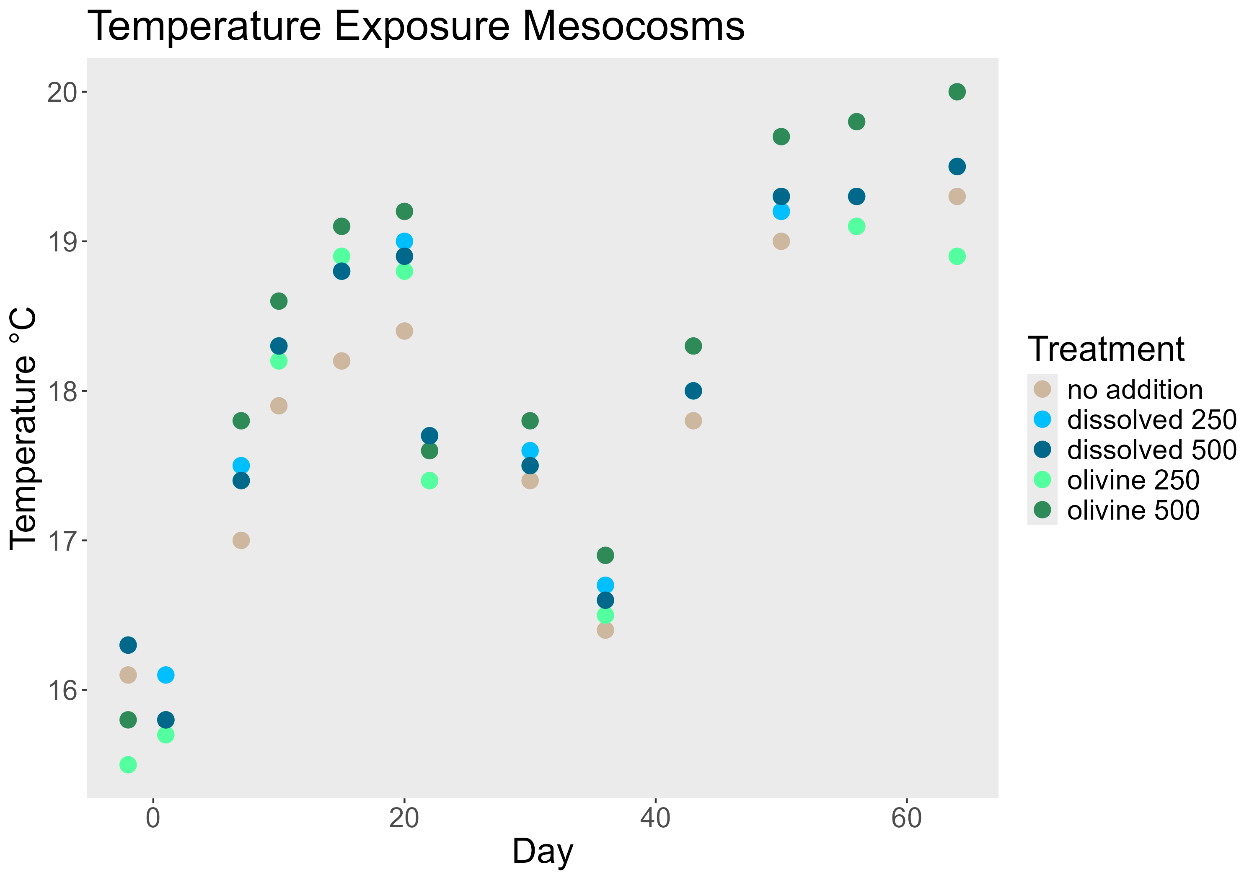


**Supplement Figure 2:** Temperature monitoring of the exposure mesocosms during the experiment. Points depict single measurements. The x-axis shows the day of the experiment, while the y-axis shows the measured temperature.


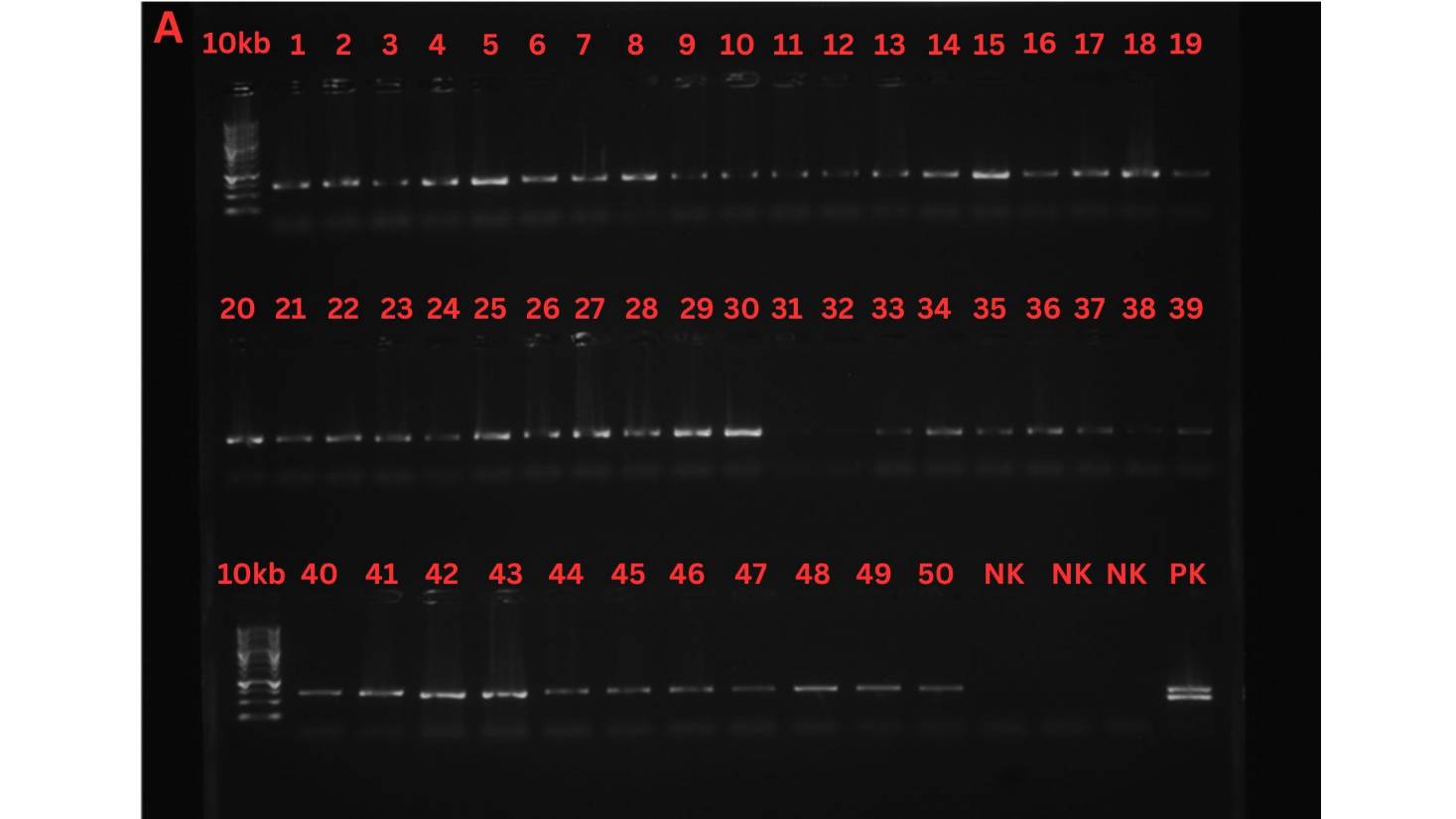


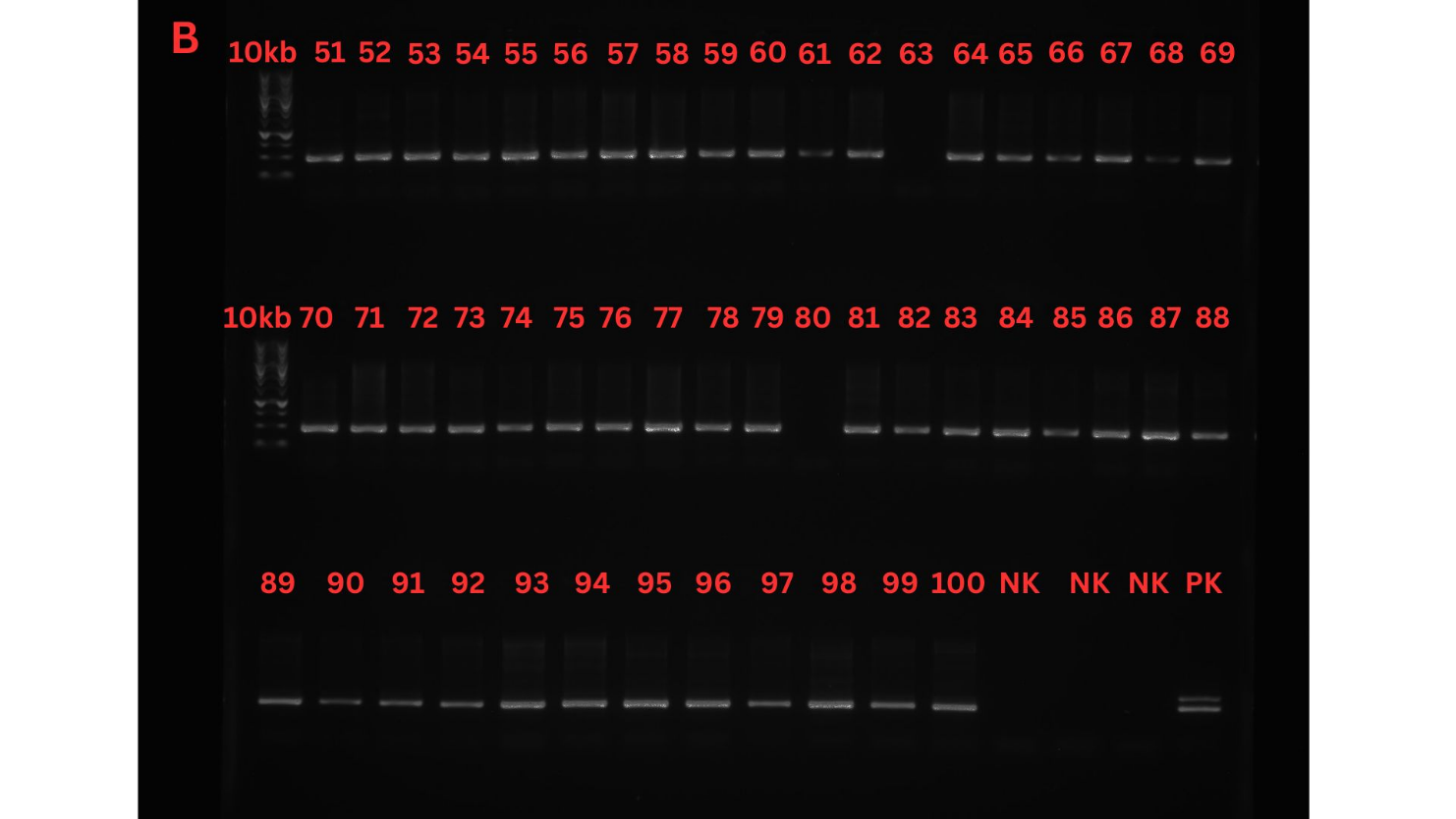


**Supplement Figure 3:** Gel pictures of PCR products from samples taken during the experiment. The indexes for A and B stand for A = Gill samples and B = Water and Food samples. The numbers indicate the sample ID used in the meta data file. The other labels mean as follows: 10kb= marker standard used to quantify the length. NK=Negative Control. PK = Positive Control


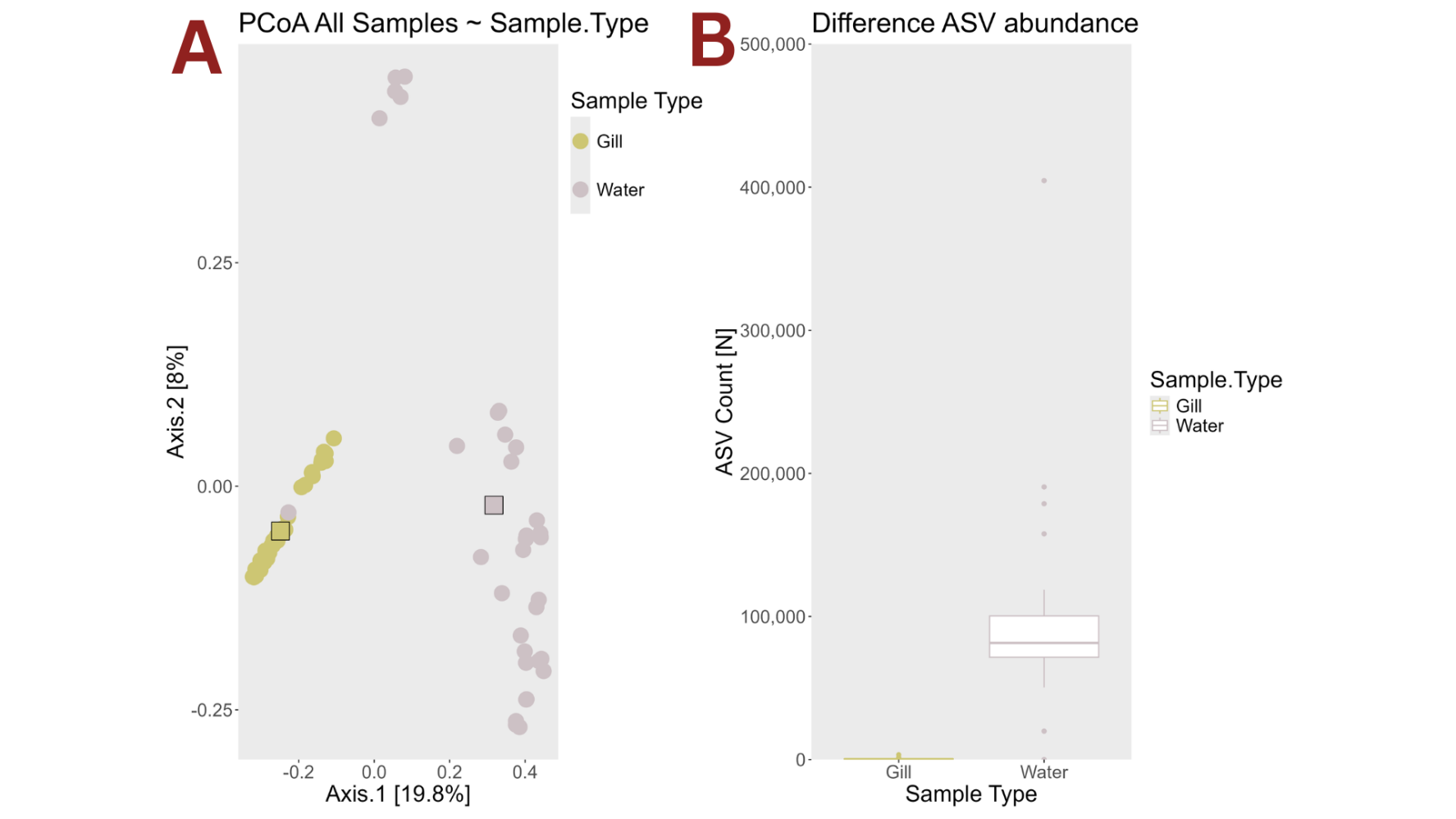


**Supplement Figure 4:**

A: PCoA of all samples sequenced color coded by the type of the sample taken. The round points depict microbiome sample while the squares represent the centroid of the sample type. B: Boxplot highlighting the differences in ASV counts among the sample types. Median value of ASVs are: Water = 81548, Gill =234. N = Absolut count values of ASVs. In the visualization the boxplot containing the gill samples and their ASVs counts are almost not visible due to the high difference to the other sample types. Gills n = 46; Water n = 33

**Supplement table 1:** Table with the specification for the PCR conducted for amplification of the 16s rRNA gene.

| Master mix | |  | Cycling Conditions | | |  |
| --- | --- | --- | --- | --- | --- | --- |
| Component | Amount |  | **Step** | **Temperature** | **Duration** | **Cycles** |
| KAPA HiFi HotStart ReadyMix | 12.5 µL |  | Pre-denaturation | 96°C | 60 s | X 1 |
| Primer 515F-Y (1µM) (5′-GTGYCAGCMGCCGCGGTAA) | 2.5 µL |  | Denaturation | 96°C | 15 s | X25 |
| Primer 926R (1µM) (5′-CCGYCAATTYMTTTRAGTTT) | 2.5 µL |  | Annealing | 55°C | 30 s | X25 |
|  |  |  | Extension | 70°C | 90 s | X25 |
| Template DNA | 5 ng in 2.5µL |  | Final Hold | 8°C | Indefinite | X 1 |
|  |  |  |  |  |  |  |

**Supplement Table 2:** Differences between targeted and reached alkalinity levels for different alkalization types. The column “Difference type” indicates the difference in alkalinity between the two treatment types. Negative values mean that the *dissolved* alkalization reached higher alkalinity than *olivine*-based alkalization. Quotation marks (〃) indicate values identical to the entry above.

| **Day** | **Treatment** | **pH** | **Delta Alkalinity** | **Alkalinity measured** | **Target levels** | **Difference type** | **Difference target** |
| --- | --- | --- | --- | --- | --- | --- | --- |
| -2 | Olivine 250 | 8.14 | 237.03 | 2,565.16 | 250.00 | 30.16 | -12.97 |
| -2 | Diss. 250 | 8.15 | 206.87 | 2,538.82 | 〃 |  | -43.13 |
| -2 | Olivine 500 | 8.21 | 395.25 | 2,719.63 | 500.00 | -45.08 | -104.76 |
| -2 | Diss. 500 | 8.25 | 440.32 | 2,772.54 | 〃 |  | -59.68 |
| 20 | Olivine 250 | 8.11 | 257.54 | 2,628.46 | 250.00 | -5.27 | 7.54 |
| 20 | Diss. 250 | 8.10 | 262.81 | 2,637.11 | 〃 |  | 12.81 |
| 20 | Olivine 500 | 8.13 | 454.52 | 2,835.47 | 500.00 | -37.12 | -45.48 |
| 20 | Diss. 500 | 8.14 | 491.64 | 2,857.81 | 〃 |  | -8.36 |
| 41 | Olivine 250 | 8.09 | 220.91 | 2,608.72 | 250.00 | -18.64 | -29.09 |
| 41 | Diss. 250 | 8.09 | 239.55 | 2,628.29 | 〃 |  | -10.45 |
| 41 | Olivine 500 | 8.14 | 392.37 | 2,783.57 | 500.00 | -75.79 | -107.64 |
| 41 | Diss. 500 | 8.12 | 468.16 | 2,860.42 | 〃 |  | -31.84 |

**supplement table 3: Table of chemical additives for dissolved alkalinity treatments.** Values are based on a mesocosm volume of 540 L. Columns represent two dissolved alkalinity target levels, while rows indicate the corresponding chemical amounts added to the mesocsms.

|  | *Dissolved 250* | *Dissolved 500* |
| --- | --- | --- |
| Weight of NaOH (g) | 5.40 | 10.81 |
| Weight of CaCl2*2H20 (g) | 9.93 | 19.86 |
|  |  |  |
